# The impact of intrinsic muscle properties on simulated reaching performance

**DOI:** 10.1080/10255842.2022.2089022

**Published:** 2022-06-30

**Authors:** Tiina Murtola, Christopher Richards

**Affiliations:** Department of Comparative Biomedical Sciences, Royal Veterinary College, London, UK

**Keywords:** Hill-type muscle model, activation dynamics, neuromuscular control, co-contraction, MuJoCo

## Abstract

Musculoskeletal modelling is used widely for studying limb motion and its control, but simulation outcomes may depend heavily on the underlying muscle model used. The aim of this study was to investigate how intrinsic muscle properties affect reaching movements in a simple upper limb model. The simulations suggest that more realistic, higher-order activation dynamics requires longer prediction from a forward model and gives rise to a higher level of unplanned co-contraction than simple activation models. Consistent with prior work, muscle force-length-velocity properties stabilised and smoothed limb movements and furthermore helped promote accurate reaching performance with the high-order activation model.

## 1. Introduction

The vertebrate limb is an intricate system whose coordinated movement emerges from interactions among its components, including muscles, connective tissues, and nervous system, as well as the environment (i.e. contact forces). How the nervous system coordinates limb movements has been studied from various perspectives, including motor control and planning (e.g. Kawato 1999), learning (e.g. Shadmehr and Mussa-Ivaldi 1994), and multi-body dynamics (e.g. Hollerbach and Flash 1982). As the component interactions are challenging to observe directly, musculoskeletal modelling allows the simulation of limb motion involving multiple joints and muscles (Arnold et al. 2000; Kargo and Rome 2002; Holzbaur et al. 2005; Delp et al. 2007). Hence, musculoskeletal models are well suited for predictive simulations (e.g. for surgery outcomes; Saul et al. 2003; Falisse et al. 2020 ), isolation of the features most crucial for behaviour (e.g. Kargo and Rome 2002; Falisse et al. 2020) or exploration of a parameter space to establish how muscle properties influence limb performance (e.g. hopping height, stability and robustness; Haeufle et al. 2012).

Musculoskeletal modelling owes many of its strengths to the simplicity of the underlying Hill-type model which is based on three experimentally measured physiological properties. First, muscle force is primarily determined by the time-varying active state which rises and falls according to the frequency of motor neuron excitation (Hill 1949; see also McMahon 1984). Second, muscle force depends on the force-length (FL) relation (Gordon et al. 1966) as well as, third, on the force-velocity (FV) relation (Hill 1938). In the present study, we focus on understanding the interactions between these muscle properties in the context of reaching movements.

Despite the simplicity and generality of standard Hill-type modelling, it has several short-comings (see Millard et al. 2019). In particular, activation is difficult to capture with standard first order models (i.e. where the first time derivative of activation is a function of the current activation and excitation) as these models neglect features shown experimentally to be important in vertebrate muscle, especially in twitches and brief contractions. First, standard models do not capture the brief delay between excitation and onset of force (i.e. electromechanical coupling delay; see Norman and Komi 1979). Second, although the twitch peak amplitude and relaxation rate can be tuned using time constants, the peak is instantaneous and its timing fixed. Finally, muscle force can continue to rise after excitation returns to zero, as seen, for example, in typical twitch responses, but in standard models, force begins to decay when excitation ceases (Lee et al. 2011). To overcome these limitations, higher-order models (i.e. higher derivatives or multiple first order equations; for example, Lee et al. 2011), have been proposed to better approximate physiologically realistic activation and adequately reproduce measured forces (Lee et al. 2013). Although the standard activation model might be sufficient for simulations using optimisation to derive muscle coordination patterns (e.g. Dembia et al. 2020), we expect that musculoskeletal modelling with feedback control could be sensitive to the intrinsic latencies in higher-order models.

Previous simulation studies using standard or no activation models suggest that FL and FV properties increase stability (van Soest and Bobbert 1993; van der Burg et al. 2005; Haeufle et al. 2010, 2012) and may reduce agonist-antagonist co-contraction (Wagner and Blickhan 2003). Sensitivities and behavioural consequences of alternative standard activation models (Rockenfeller et al. 2015; Bayer et al. 2017) and FV and FL curves have also been studied previously (Bayer et al. 2017), but the effect of combining higher-order activation models with FL and FV properties remains unexplored except for smoothing effects in single muscle contractions (Krylow and Rymer 1997). Hence, in the present study, we aim to use musculoskeletal modelling to investigate how these three intrinsic muscle properties interact to influence reaching. Additionally, we explore whether a simple predictive feedback controller is sufficient to compensate for the challenges posed by controlling a system with increasingly realistic muscles.

## 2. Methods

### 2.1. Models

**Figure 1.**
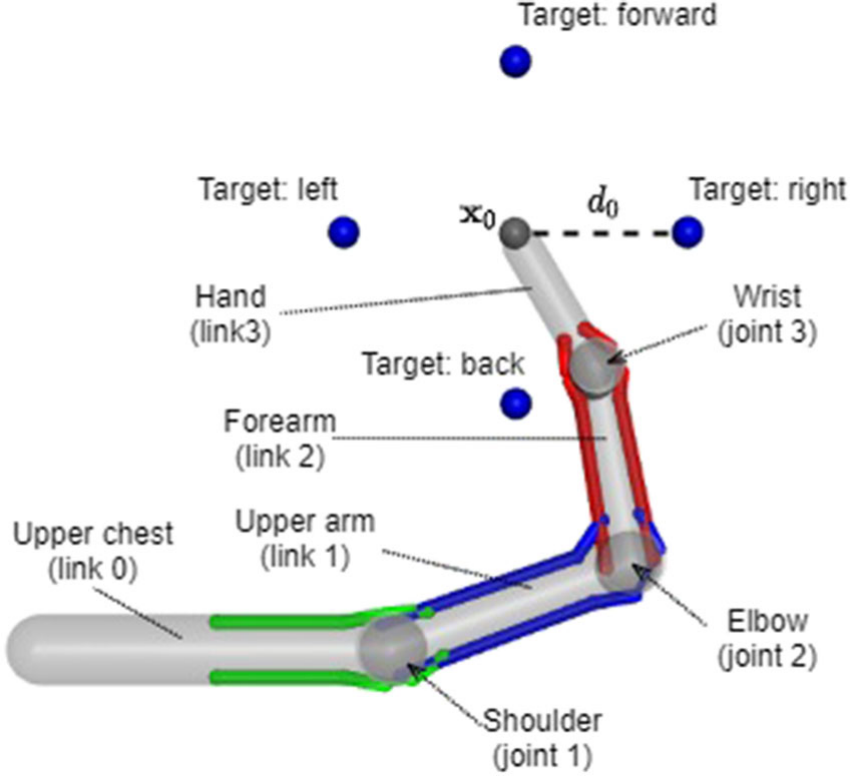
The arm model consisting of four links, joined by three hinge joints, and controlled using three agonist-antagonist muscle pairs (coloured by joint). Tracked site endpoint of the hand link is shown as a gray sphere and the position of targets by blue spheres.

We designed a simplified model (Figure 1) which grossly matches human anatomy, but without anatomical details unlikely to affect general principles governing muscle-powered reaching. This planar, three degree-of-freedom model consists of a stationary ground link (upper chest) and three distal links (upper arm, forearm and hand). The links are connected with three hinge joints (shoulder, elbow and wrist), enabling the links to move in the horizontal plane. The joint angles $q(t)\in Q\subset R^{3}$ are limited to the range *Q*. The distal endpoint of the hand link is tracked for the reaching movements, and its position is denoted $x(t)\in R^{2}.$ The dimensions of the limb and ranges of the joints are shown in Table 1.

**Table 1.** Parameters for the arm model.

Parameter	Upper chest	Upper arm/Shoulder	Forearm/Elbow	Hand/Wrist
Link length (m)	0.44	0.32	0.25	0.18
Link mass (kg)	3.1	1.9	1.1	0.41
Wrapping radius (m)	–	0.045	0.041	0.035
Feasible joint range (deg.)	–	(−20,120)	(5, 120)	(−60,70)
Initial joint angle (deg.)	–	20	80	20
Flexor isometric strength (N)	–	1500	1300	500
Extensor isometric strength (N)	–	1500	1000	300

Six muscles, indexed j=1,…,6 and arranged in three agonist-antagonist pairs, are connected to the links. Each muscle pair crosses a single joint, with cylindrical wrapping surfaces at the joints ensuring roughly constant moment arms. Hence, each joint has one flexor and one extensor muscle^1^.

The dynamics of the model are governed by

(1) $$M\ddot{q}+c-f_{c}=T,$$

where $M=M(q)\in R^{3\times 3}$ is the inertia matrix of the model, $c=c(q,\dot{q})\in R^{3}$ contains Coriolis effects, $f_{c}=f_{c}(q)\in R^{3}$ contains constrain forces in joint space, and $T\in R^{3}$ are joint torques arising from muscle contraction. The joint torques can be further decomposed into

(2) T=R F a,

where $R=R(q)\in R^{3\times 6}$ is a matrix of moment arms, $F=F(l,\dot{l})\in R^{6\times 6}$ is a diagonal matrix of maximal muscle forces producible at muscle length $l(q)\in R^{3}$ and contraction velocity $\dot{l}=\frac{d}{dt}l(q),$ and $a=a(u)\in I^{6},$ where I=[0,1], is the muscle activation state in response to an excitation $u=u(t)\in I^{6}.$ Passive force production in the muscles is not included as its contribution in the tasks performed (see below) is minor due to the small changes in muscle lengths.

#### 2.1.1. Muscle models

**Figure 2.**
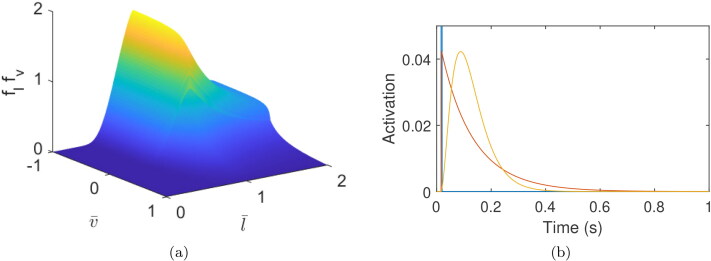
Muscle properties: Force-length-velocity characteristics (a) and activation response to a unit excitation impulse (b). In (b), excitation/instantaneous activation model shown in blue (amplitude cut off for plotting), first order activation model in red and third order activation model in yellow.

The force output of muscles in Equation (2) depends on intrinsic muscle properties via **F** and **a**. Two alternatives for the FL and FV properties represented by **F** are considered. In the first case, force generation is independent of muscle length and velocity, that is, the diagonal elements of **F** are given by

(3) $$F_{jj}=f_{\max,j},$$

where $f_{\max,j}$ is the isometric strength of muscle *j*. In the second case, the potential force output of muscle depends on its normalised length ${\tilde{l}}_{j}=l_{j}/l_{0,j},$ where $l_{0,j}$ is the optimal length of the muscle, and normalised contraction speed ${\tilde{v}}_{j}=\dot{l}/l_{0,j}/v_{\max},$ where *v_max_* is the maximum contraction speed. Namely,

(4) $$F_{jj}=f_{\max,j}f_{l}({\tilde{l}}_{j})f_{v}({\tilde{v}}_{j}),$$

where

(5) $$f_{l}(\tilde{l})=e^{-{\left|\frac{{\tilde{l}}^{b_{2}}-1}{b_{3}}\right|}^{b_{1}}}$$

with shape parameters $b=(b_{1},b_{2},b_{3}),$ and

(6) $$f_{v}(\tilde{v})=\{\begin{array}{cc} \frac{1-\tilde{v}}{1+d_{1}\tilde{v}}, & \text{if}\;\tilde{v}>0\\ d_{2}-\frac{\left(d_{2}-1)(1+\tilde{v}\right)}{1-d_{3}\tilde{v}}, & \text{otherwise} \end{array}$$

with shape parameters $d=(d_{1},d_{2},d_{3}).$ This model combines the separate FL and FV relationships into a single force-length-velocity (FLV) function, $f_{v}f_{l},$ which is shown in Figure 2.

The $f_{\max,j}$ values of the muscles are listed in Table 1. Given our model’s simplicity, human data cannot be used directly. Instead, $f_{\max,j}$ values are selected so that both the peak forces and torques are in the correct order of magnitude (cf. e.g. Holzbaur et al. 2005). The lengths $l_{0,j}$ are assumed to correspond to the initial position of the arm (see Table 1), and the remaining muscle model parameters are listed in Table 2.

**Table 2.** Muscle model and task parameters.

Parameter	Symbol	Value
FL shape parameters	**b**	(1.3, −1.3, 0.53)
FV shape parameters	**d**	(4, 1.8, 30.24)
Max. contraction speed	*v_max_*	1.6 muscle lengths/s
O1-AM time constants	$\alpha_{O1}$	(94.5, 65) ms
O3-AM time constants	$\alpha_{O3}$	(18, 40, 25) ms
O3-AM nonlinearity coeff.	$\beta_{O3}$	(0.6, 0.8, 0.7)
Target distance	*d*_0_	0.2175 m
Planned movement duration^1^	*T_d_*	1.03 s
Simulation duration	*T_max_*	4 s
Error tolerance	*ϵ_tol_*	1 mm

^1^ Computed as $1.712d_{0}^{1/3}$ (Miranda et al. 2018)

The activation state **a** determines what fraction of the muscles’ force generation potential is in use at any point in time. Three different models for mapping from excitation to activation are considered (Figure 2b).

1. In the instantaneous activation model (IAM), the activation state tracks the excitation, that is,
  (7) a=u.
2. In the first order activation model (O1-AM), muscle activation dynamics are determined by the commonly used (e.g. in OpenSim (Delp et al. 2007) and MuJoCo (Todorov et al. 2012)) nonlinear differential equation
  (8) $$A(a,u)\dot{a}+a=u,$$

where A(a,u) is a diagonal matrix containing time parameters which differ for activation and deactivation phases according to

(9) $$A_{jj}=\{\begin{array}{cc} \alpha_{\mathrm{act}}(0.5+1.5a_{j}) & \text{if}\;u_{j}>a_{j}\;\text{and}\\ \alpha_{\mathrm{deact}}/(0.5+1.5a_{j}) & \text{otherwise}. \end{array}$$

The time scale of the activation dynamics hence depend on $\alpha_{O1}=(\alpha_{\mathrm{act}},\alpha_{\mathrm{deact}}).$

In the third order activation model (O3-AM), the activation state is determined by a cascade of three first-order differential equations (Lee et al. 2011),
  (10) $$\begin{array}{cc} {\dot{a}}_{1}+\frac{1}{\alpha_{1}}\left(\beta_{1}+[1-\beta_{1}]u\right)a_{1} & =\frac{1}{\alpha_{1}}u,\\ {\dot{a}}_{2}+\frac{1}{\alpha_{2}}\left(\beta_{2}+[1-\beta_{2}]a_{1}\right)a_{2} & =\frac{1}{\alpha_{2}}a_{1},\\ \dot{a}+\frac{1}{\alpha_{3}}\left(\beta_{3}+[1-\beta_{3}]a_{2}\right)a & =\frac{1}{\alpha_{3}}a_{2}, \end{array}$$
  where $a_{1}$ and $a_{2}$ are intermediate state variables, $\alpha_{O3}=(\alpha_{1},\alpha_{2},\alpha_{3})$ are time constants and $\beta_{O3}=(\beta_{1},\beta_{2},\beta_{3})$ are nonlinearity coefficients.

The parameter values of O1-AM and O3-AM (Table 2) were selected as follows. First, the parameters of the O3-AM were adjusted from the values of Lee et al. (2011) to achieve realistic time scale for human muscles (cf. Belanger and McComas 1985) resulting in twitch time-to-peak of 70 ms, peak amplitude of 0.0423, and half-relaxation time of 82 ms. Second, the time constants of the O1-AM were set so that the twitch peak amplitude and half-relaxation time matched O3-AM.

Five model variations are constructed out of the above muscle models. Constant and nonlinear FLV modulation is combined with O1-AM (models named FMAX + O1 and FLV + O1, respectively) as well as with O3-AM (models FMAX + O3 and FLV + O3), and a reference model is constructed by combining constant force modulation with IAM (model FMAX). The muscle models and their constituent equations are summarised in Table 3.

**Table 3.** Summary of the five muscle models used in this study and the force generation and activation dynamics equations used in each model.

Model	Activation	Force generation
FMAX	IAM, Equation (7)	Constant, Equation (3)
FMAX + O1	O1-AM, Equation (8)	Constant, Equation (3)
FLV + O1	O1-AM, Equation (8)	Nonlinear FLV, Equations (4)–(6)
FMAX + O3	O3-AM, Equation (10)	Constant, Equation (3)
FLV + O3	O3-AM, Equation (10)	Nonlinear FLV, Equations (4)–(6)

#### 2.1.2. Model control

**Figure 3.**
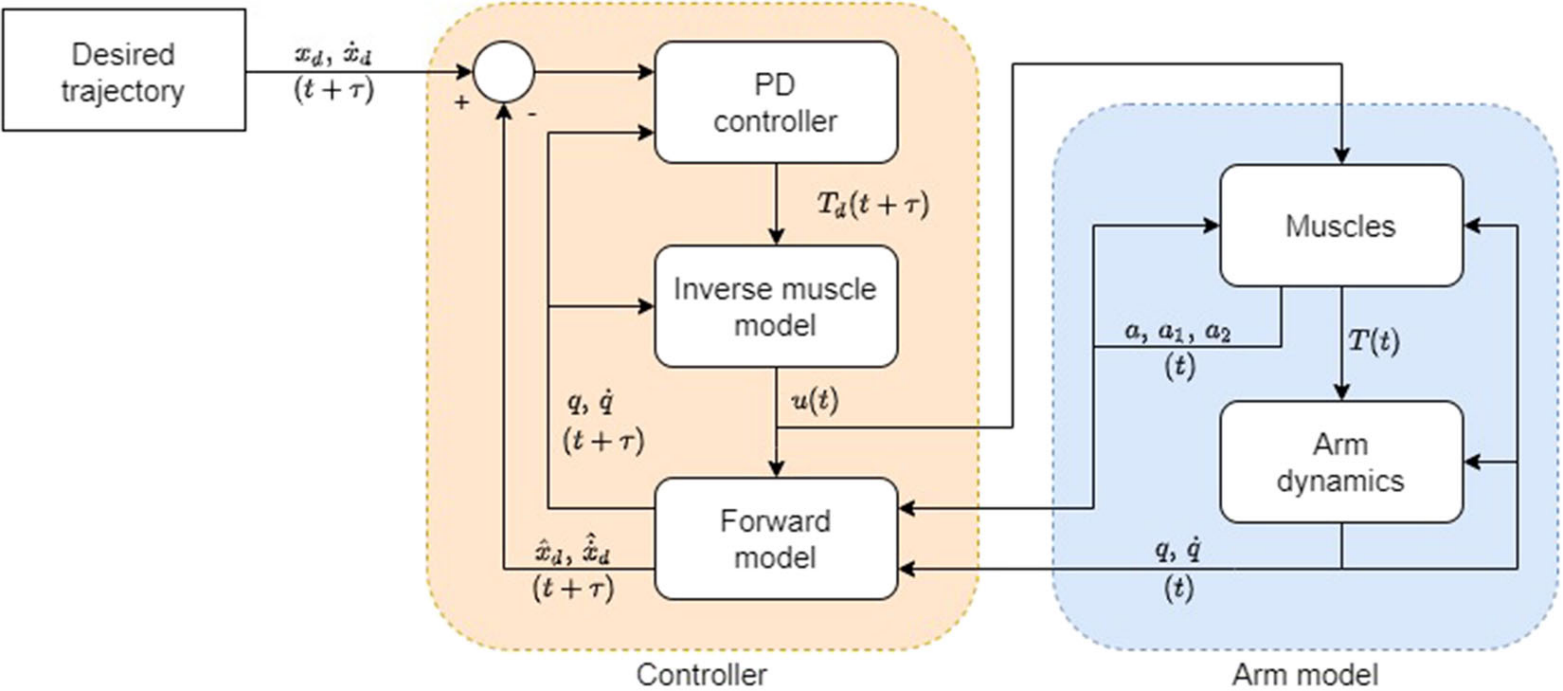
Schematic representation the arm model and its control system.

For this study, reaching is formulated as tracking a desired trajectory $x_{d}=x_{d}(t).$ Figure 3 shows schematically how **u** is computed using prediction to mitigate the destabilising effects of the delays arising from muscle activation dynamics. At every time step *t*, a forward model (copy of the current model) is used to predict the state of the system at t+τ (with the excitation **u** kept constant between *t* and t+τ). Desired joint torques, $T_{d}(t+\tau ),$ are computed from the error between desired and predicted states using a PD controller,

(11) $$T_{d}=K_{p}{\hat{J}}^{†}(x_{d}-\hat{x})+K_{v}{\hat{J}}^{†}({\dot{x}}_{d}-\hat{\dot{x}}),$$

where $K_{p},K_{v}\in R_{+}^{3\times 3}$ are diagonal gain matrices, ${\hat{J}}^{†}=J^{†}(\hat{q})\in R^{3\times 2}$ is the pseudo-inverse of the Jacobian at the predicted state, and $\hat{x}$ and $\hat{\dot{x}}$ denote the predicted endpoint position and velocity, respectively. The model dependent values of $K_{p},\;K_{v},$ and *τ* are obtained by numerical optimisation (see Section 2.2.2).

An inverse muscle model is used to convert $T_{d}(t+\tau )$ into excitation at the current time step, u(t), using two assumptions. First, it is assumed that the true activation dynamics of Equations (7)–(10) can be approximated with a constant delay of duration *τ* so that a(t+τ)≈u(t). This assumption enables an approximate inversion of the activation dynamics without considering the multiple past excitation patterns which can give rise to any activation state. Second, it is assumed that there is no co-excitation (i.e. simultaneous non-zero excitation of both the flexor and extensor of a joint). This removes redundancy from the choice of muscles by setting excitation the antagonists (determined by direction of muscle torque relative to $T_{d}$) to zero. Based on these two assumptions, Equation (2) can be rearranged to compute **u** in two parts

(12) $$u_{G}(t)=a_{G}(t+\tau )={\left(R_{G}\;F_{G}\right)}^{-1}T_{d}(t+\tau )\;\text{and}\;u_{N}(t)=0,$$

where $u_{G}$ and $a_{G}$ contain the elements of the excitation and activation state corresponding to the agonist muscles, $R_{G}$ and $F_{G}$ contain the columns of **R** and **F** corresponding to the agonists, and $u_{N}$ contain the elements of the excitation corresponding to the antagonists. If the desired torque exceeds an agonists capacity to produce torque, the corresponding element of $u_{G}$ is set to one.

### 2.2. Simulations

#### 2.2.1. The task set and performance metrics

Four targets are placed at a distance *d*_0_ around the initial endpoint position $x_{0}$ (see Figure 1). The targets are referred to as right, forward, left and back based on their position relative to $x_{0}.$ The reaching tasks are completed sequentially, returning the model to its initial state between each reach. Denoting the position of the target $x_{t},$ the trajectory of the reach is planned as

(13) $$x_{d}(t)=x_{0}+\left(x_{t}-x_{0}\right)\int_{0}^{t}v(s)ds,$$

where the endpoint speed,

(14) $$v(t)=\{\begin{array}{c} \frac{1}{T_{p}}\left[30{\left(\frac{t}{T_{p}}\right)}^{4}-60{\left(\frac{t}{T_{p}}\right)}^{3}+30{\left(\frac{t}{T_{p}}\right)}^{2}\right],\;\text{if}\;t<T_{p},\\ 0\;\text{otherwise}, \end{array}$$

is based on minimising jerk (Flash and Hogan 1985) and *T_p_* is the planned duration of the movement. For ease of discussion, the first part of the movement with $t<T_{p}$ is hereafter referred to as the planned phase and the second part with $t\geq T_{p}$ is referred to as the stabilisation phase.

The performance of the models is measured using two errors. First, we define movement error, *e_mv_*, for a reach to be the average deviation from planned trajectory

(15) $$e_{mv}=\frac{1}{T_{\max}}\int_{0}^{T_{\max}}||x_{d}(t)-x(t)||dt,$$

where *T_max_* is the duration of the simulation. Because accurate tracking is a more constraining task than simple reaching, a second error characterising the higher-level outcome, namely deviation from a stable final position at the target, is also used. This is the stabilisation error

(16) $$e_{st}=\frac{1}{T_{\max}-t_{\mathrm{succ}}}\int_{t_{\mathrm{succ}}}^{T_{\max}}||x_{d}(t)-x(t)||dt,$$

where *t_succ_* is the time when the target is first reached successfully in the sense of $||x_{d}(t_{\mathrm{succ}})-x(t_{\mathrm{succ}})||\leq \epsilon_{\mathrm{tol}}.$ If the endpoint does not come within the tolerance *ϵ_tol_* of the target during the simulation, *t_succ_* is set to zero so that *e_st_* = *e_mv_*.

Both *e_st_* and *e_mv_* are computed separately for each model, set of control parameters, and target. To compare the performance of models and control parameters, each error is averaged over the four target locations, and these average stabilisation and movement errors are denoted ${\bar{e}}_{st}$ and ${\bar{e}}_{mv},$ respectively.

#### 2.2.2. Numerical implementation and control parameters

The forward dynamics simulations with the model are carried out using MuJoCo (Todorov et al. 2012) with simulation time steps of 2 ms. Source code for the implementation is available at https://github.com/tmmurtola/reaching-arm-model.

The performance of the models depends on the feedback gains $K_{p}$ and $K_{v}$ and the prediction time *τ*, which were optimised numerically to find the best achievable performance of each model variation. Two optimisations are carried out for each model: one minimising ${\bar{e}}_{st}$ and another minimising ${\bar{e}}_{mv}.$ The optimisations are implemented in Matlab with mixed-integer genetic algorithm (nb. *τ* is a discrete number of simulation time steps).

## 3. Results

### 3.1. Reaching performance

**Figure 4.**
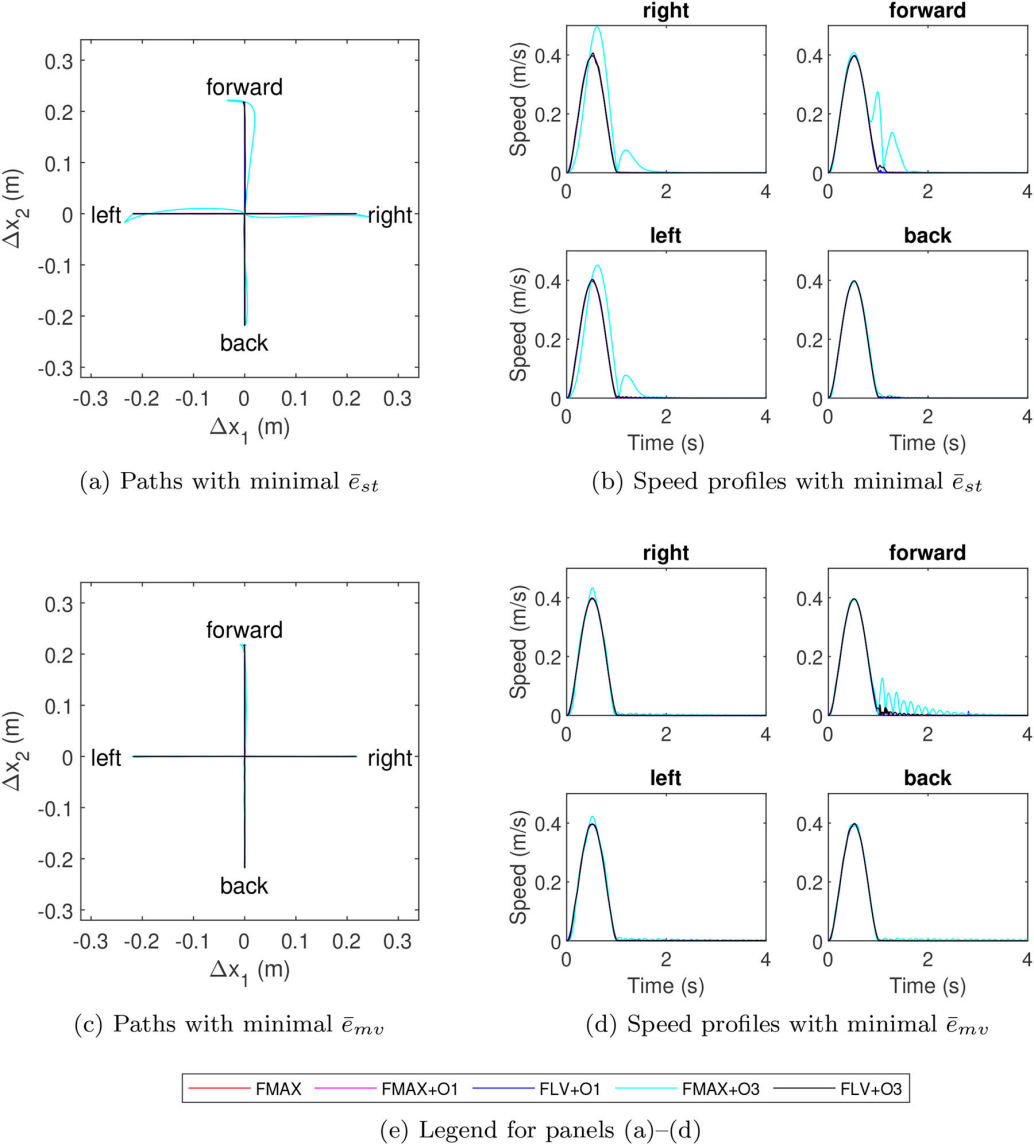
Reaching paths and endpoint speed profiles the four target locations under the two optimisation criteria. Note that due to similarity of the data, FLV + O3 largely obscures FMAX, FMAX + O1 and FLV + O1 trajectories.

Highly accurate tracking of planned trajectories was achieved for all models except FMAX + O3 (Figure 4 and Table 4), with ${\bar{e}}_{st}$ below 58 µm and ${\bar{e}}_{mv}$ below 199 µm for both optimisation criteria. The paths traced by the four models (FMAX + O3 excluded) were virtually identical, except for very slight deviations for the forward reaches. Similarly, the four models adhered well to the planned speed profile, except for small decaying oscillations at the start of the stabilisation phase exhibited by all models.

**Table 4.** Feedback gains and prediction times obtained using the two optimisation criteria and the corresponding average errors.

Model	$K_{p}$ (Nm/rad)			$K_{v}$ (Nms/rad)			*τ* (ms)	${\bar{e}}_{st}$ (mm)	${\bar{e}}_{mv}$ (mm)
Minimised ${\bar{e}}_{st}$									
FMAX	5582.22	2050.08	7541.40	80.81	0.23	0.78	0	0.007	0.019
FMAX + O1^1^	6920.38	328.13	316.41	585.00	45.85	6.89	4	0.010	0.114
FLV + O1^1^	7527.00	561.63	223.45	553.54	54.70	7.48	4	0.009	0.113
FMAX + O3	38.33	0.18	2.96	11.12	0.23	0.24	46	0.080	4.451
FLV + O3	192.97	671.47	289.37	19.32	7.70	2.14	42	0.023	0.199
Minimised ${\bar{e}}_{st}$									
FMAX	4342.35	5154.67	1622.29	71.27	0.37	0.36	0	0.007	0.009
FMAX + O1^1^	6919.67	325.00	400.00	582.36	45.63	7.60	4	0.010	0.114
FLV + O1	7670.65	513.68	329.60	546.80	56.62	7.93	4	0.009	0.112
FMAX + O3	67.89	3.36	15.03	21.44	1.71	0.35	36	0.297	0.702
FLV + O3	853.76	743.07	270.68	39.11	12.74	1.73	42	0.058	0.133

^1^ Multiple similar optima with different gains and *τ* in the range 0–4 ms were found.

The performance of FMAX + O3 differed from the others notably. Successful reaching could only be achieved with low gains, and the resulting movements either exhibited curved paths with multi-peaked endpoint speeds (under ${\bar{e}}_{st}$ minimisation) or visible oscillations during the stabilisation phase (under ${\bar{e}}_{mv}$ minimisation). These movement features were particularly noticeable during forward reaches. The optimal control parameters listed for FMAX + O3 (Table 4) required multiple optimisation attempts, suggesting that an optimum producing better performance could exist but is difficult to find. Nonlinear FLV modulation appeared to help achieve controllability with O3-AM and make the optimisation task easier, whereas the role of FLV modulation was small with O1-AM. The optimal gains for FMAX + O1 and FLV + O1 are similar, and while including FLV modulation in the muscle model improves performance, the decreases in both ${\bar{e}}_{st}$ and ${\bar{e}}_{mv}$ are small.

The optimal *τ* values differed between activation models. IAM required no prediction, while the models with O1-AM produced best outcomes with *τ* between 0 and 4 ms. For models with O3-AM, longer prediction, between 36 and 46 ms, was needed. The fact that ranges of optimal *τ* were observed, rather than a single value for each model, reflect the fact that reaching performance is determined by the combination of prediction time and feedback gains, so that slight variations in one value can be compensated for by changes in the others.

### 3.2. Joint torque and co-contraction patterns

**Figure 5.**
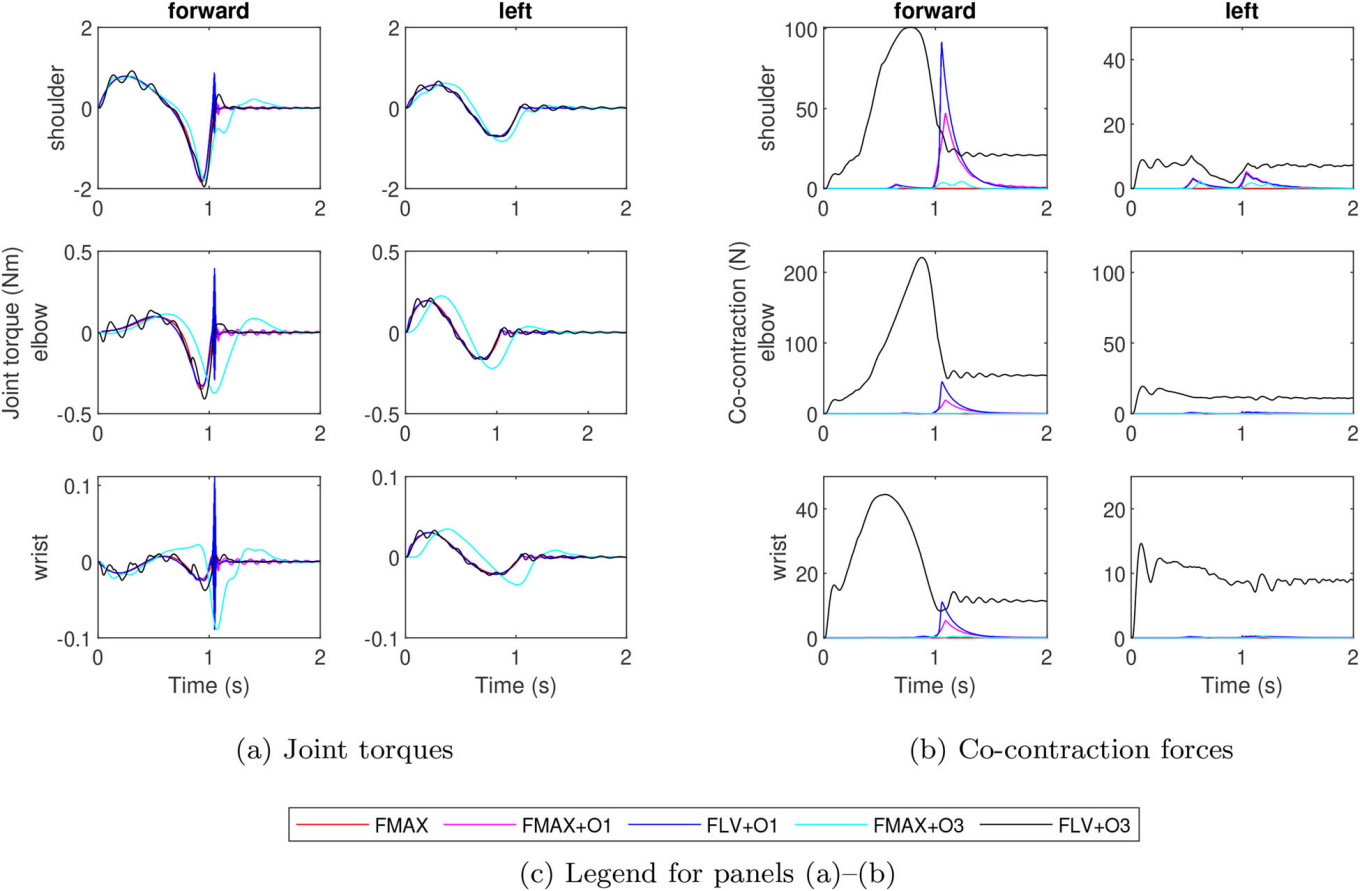
Joint torque and co-contraction force in muscle pairs crossing the shoulder (top row), elbow (middle row), and wrist (bottom row) for reaches for forward and left targets. Control parameters producing minimum stabilisation error were used. Positive torque corresponds to flexion and negative to extension in (a).

While the choice of muscle model does not appear to affect the ability of the arm to complete the reaching tasks accurately, the patterns of muscle contraction vary between models. Figure 5 illustrates this by showing the net torques and co-contraction forces by joint for the reaches towards the forward and left targets for simulations with minimised ${\bar{e}}_{st}.$ The joint torques, given by Equation (2), follow similar overall patterns across the models but the level of oscillations varies: FLV + O3 show oscillations throughout both movements while the models with O1-AM only show high-frequency oscillations at the end of the planned phase of the forward reach, and FMAX shows virtually no oscillations. While the shapes of the joint torque patterns for FMAX + O3 are similar to the other models, these patterns appear delayed in time, especially for the elbow and wrist joints. This delay in torque generation is likely due to the low feedback gains as the effect was not visible with the higher gains corresponding to minimal ${\bar{e}}_{mv}.$

In addition to the oscillations in the joint torque, the models also differ in the patterns of co-contraction force, defined here as the force exerted simultaneously by the agonist and antagonist muscles. The baseline model, FMAX, cannot produce co-contraction due to the assumption of no co-excitation. The models with O1-AM produce bursts of co-contraction when the joint torque changes direction, but the level of co-contraction in these bursts is negligible, except at the beginning of the stabilisation phase of the forward reach (Figure 5b). The co-contraction pattern of FMAX + O3 resembles those of FMAX + O1 and FLV + O1 but with low, oscillating levels of co-contraction at the beginning of the stabilisation phase. These oscillations reflect the multiple changes of joint torque directions FMAX + O3 exhibits, and the low level of co-cocontraction suggests that the curved path to which FMAX + O3 converged is more energy efficient than strict adherence to the planned trajectory.

The co-contraction in FLV + O3 differs from the other models both in amplitude and in the temporal pattern. Higher levels of co-contraction occur throughout both planned and stabilisation phases of the movement, and this trend holds for all targets as well as both optimisation criteria. In all simulations and across all joints with FLV + O3, co-contraction peaks during the planned phase of the movement before settling on a lower, sustained level during the stabilisation phase. The forward and left reaches display two extremes of this pattern (Figure 5b): co-contraction is up to three times higher during the planned phase of the forward reach than during the stabilisation phase whereas the difference between the phases is more moderate for the left reach.

### 3.3. Task-wise accuracy and co-contraction

**Figure 6.**
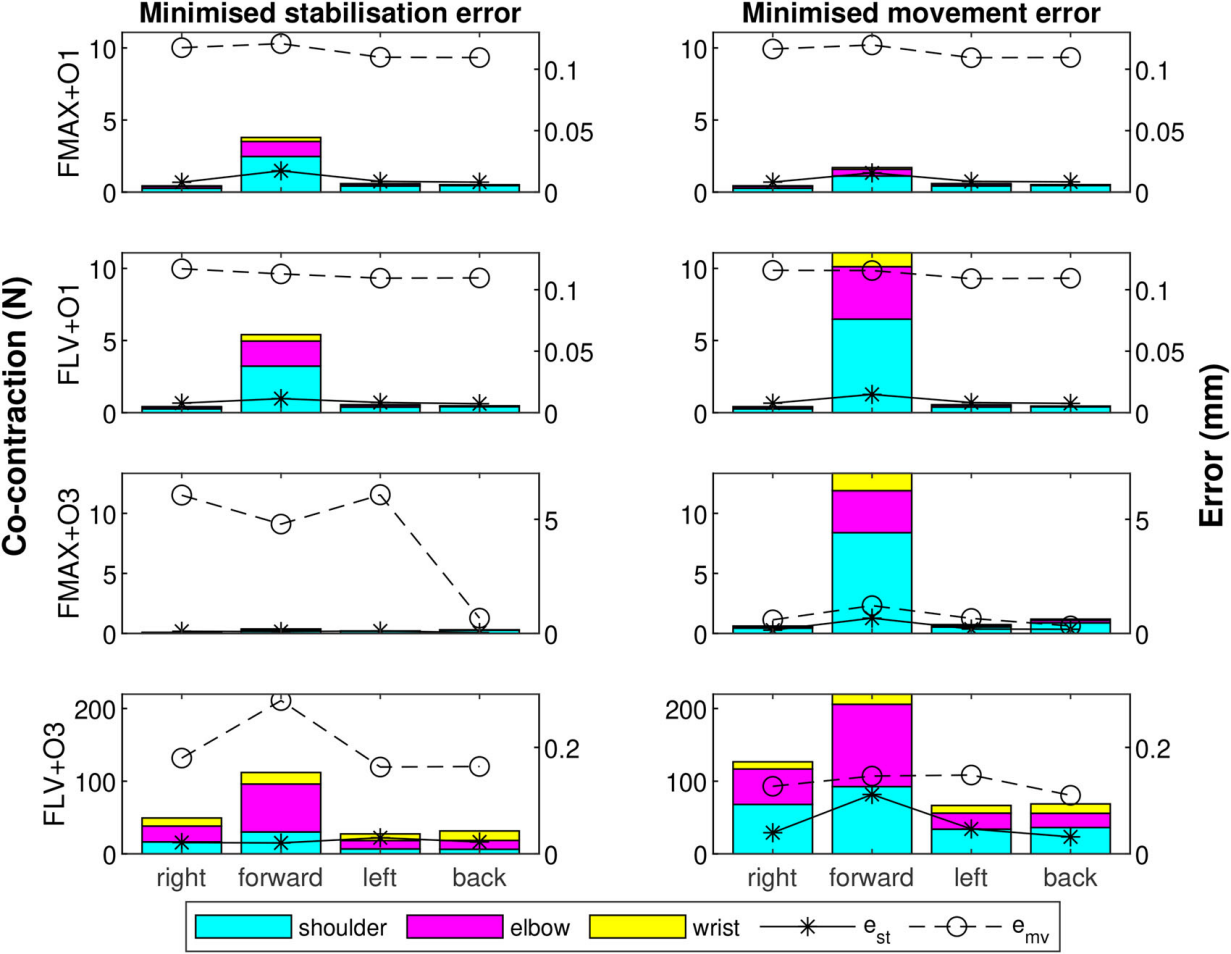
Average co-contraction force (bars, primary y-axis) and stabilisation and movement errors (lines, secondary y-axis) for each target by model (FMAX excluded). Left column corresponds to simulations minimising the stabilisation error (across all four tasks) and right column to minimising the movement error.

Figure 6 summarises the average co-contraction forces for each model variation (except the co-contraction free FMAX) by target location and joint. For context, *e_st_* and *e_mv_* are also displayed for each target. As indicated by Figure 5, reaches towards the forward target require a high level of co-contraction compared to the other directions for all models. There is also a tendency for minimisation of ${\bar{e}}_{mv}$ to lead to higher co-contraction than when ${\bar{e}}_{st}$ is minimised, although the opposite is true for FMAX + O1. Despite these trends, the average levels of co-contraction are negligible compared to the isometric strengths of the muscles for all models except FLV + O3 and even for FLV + O3 they remain below 12% of the isometric muscle strength.

The high co-contraction levels for the forward reaches tend to be accompanied by high *e_st_* values while directional trends in *e_mv_* are less pronounced. The reduced accuracy in forward reaches suggests that the observed high co-contraction emerges as an automatic strategy to deal with a difficult task. The difficulty of the forward reach arises from the location of the target near the edge of the arm’s workspace. It requires a nearly straight arm to reach which makes accurate control of the endpoint position harder as only the shoulder joint can be used effectively and the straight arm amplifies any errors made by the controller.

Regardless of optimisation criteria, the introduction of FLV modulation into the O1-AM causes small decreases in co-contraction across all joints and nearly all tasks. For forward reaching, however, co-contraction increases. Addition of FLV modulation into the O3-AM increases co-contraction significantly, but this is also accompanied by a large improvement in the performance of the model, with performance errors in the FLV + O3 only 3–56% of the corresponding error values in FMAX + O3. Hence, Figure 6 illustrates that FLV modulation plays an important role in stabilising the final position of the forward reach while also improving the overall tracking accuracy when O3-AM is used.

## 4. Discussion

We carried out simulations with a simplified arm model controlled via different muscle models to investigate how intrinsic muscle properties affect reaching performance; particularly, how more realistic activation dynamics affect the control of reaching. The simulations indicate that all model variations are capable of tracking a human-like trajectory to a target and accurately stabilising the endpoint position. This performance was achieved with a simple predictive feedback controller, but the controller needed to be tuned to each model version separately.

### 4.1. Activation dynamics affected prediction time, τ, and accuracy

The activation models’ effect on *τ* and accuracy were expected, as delays, such as those introduced by activation dynamics, are a known cause of instability in feedback-controlled system (Niculescu 2001). While various control strategies have been proposed to deal with delays in engineering applications (e.g. Smith 1959; Tan et al. 2001), most work has focused on transmission-type delays where control or observation signals are simply time shifted. In contrast, muscles exhibit relatively little dead time; instead, force delay arises because full activation takes time to develop and dissipate. These transient periods differ between activation models particularly when twitch, rather than tetanic, responses dominate. The optimal *τ* values observed, especially the similarly low *τ* for O1-AM and IAM and high *τ* for O3-AM, suggest further that the muscles’ initial response to fast changing excitation is a key feature for the control of the reaching tasks investigated.

The duration of predictions is an inherent variable in any forward model, but it plays a particularly large role in tracking tasks. In a biological system, different muscles may exhibit different activation dynamics depending, for example, on their muscle fibre composition (Lee et al. 2011), making the determination of *τ* challenging. However, the reaching performance of our model remained acceptable for small variations in *τ*, suggesting that an average *τ* may suffice for a collection of muscles with different activation speeds. Furthermore, the forward and inverse models within the controller do not need to be accurate separately as long as they form a sufficiently good identity mapping with the system being controlled (Jordan and Rumelhart 1992). Hence, inaccuracies in *τ* could potentially be compensated for by other structures in the controller, but for a more complex arm model, this likely requires a more complex control strategy as well.

Alternatives to the activation models compared in this study include both first order (e.g. Hatze 1978; Zajac 1989) and higher order models (e.g. Bobet et al. 1993). Comparing the exact impact of all available activation models would require exhaustive simulations, but the results presented above suggest behavioural consequences are dominated by the transient periods from excitation to force generation. Hence, we would expect similar performance and control consequences from all of the first order models due to their common limitations, and we would also expect higher-order models with similar transient behaviour to require similar re-tuning of control parameters.

### 4.2. FLV muscle properties improve reaching performance

Our findings are consistent with prior work suggesting that FLV properties function as a zero-delay feedback mechanism that resists perturbations (e.g. van Soest and Bobbert 1993; van der Burg et al. 2005) and smooths movements (Krylow and Rymer 1997) in tasks such as maximum-height vertical jumps (van Soest and Bobbert 1993) and maximum-velocity movements of a single joint (Bayer et al. 2017). However, the FLV effect observed in simulations for the O1-AM was smaller than would be expected based on literature, likely due to two factors. First, the reaches consisted of relatively short movements at moderate pace, which require a smaller range of muscle lengths and contraction velocities than movements calling for maximal velocity or force. Second, the FLV properties and parts of the model’s controller may serve overlapping functions (e.g. damping by FV properties and velocity controller). Hence, the chosen controller and the degree to which control parameters are re-optimised for different FLV characteristics can have a major effect on simulation outcomes, limiting the comparability of different studies.

The speed and range of joint movements determine how much the FV and FL gains vary during a movement. Hence, FLV modulation becomes more important in demanding reaches such as those requiring high movement speed or extreme joint excursions. The force amplification/attenuation provided by FLV is limited, however, so it is possible that for sufficiently difficult tasks, it cannot provide enough stabilisation to keep performance unaffected. While this could potentially play a role in known performance limits, such as the trade-off between movement speed and the accuracy of the final position (Fitts 1954), it is also possible that the increased stabilisation needs are met using alternative strategies, such as co-activation (Gribble et al. 2003). Further, the presence of redundant and bi- or multi-articular muscles enable switching between muscles depending on where they are on their FLV curves. We note that the relatively easy reaching tasks studied presently represent most daily reaching movements, as other strategies, such as moving and orienting the body and choosing the reaching arm, can be used to limit the need for fast or large amplitude arm movements.

### 4.3. Our simplified controller produced successful behaviour despite the challenges of realistic muscle properties

Targets were reached successfully without exact inversion of model dynamics or muscle models. The cost, however, was increased co-contraction when high-order activation was used. The observed co-contraction differs conceptually from traditional impedance control (Hogan 1984) in which co-contraction is typically viewed as a centrally planned response to factors such as unstable environment (Burdet et al. 2001), noise in control signals (Selen et al. 2005), or increased accuracy requirements (Gribble et al. 2003). In our model, co-contraction arises from the fact that the activation state cannot be turned on and off instantaneously, resulting in distinctive patterns around changes in torque directions. This co-contraction represents a virtually unavoidable baseline level, on top of which planned impedance control can be added.

The present control strategy follows the traditional approach (e.g., Flash and Hogan 1985) of separating the planning and execution of movements, although experimental evidence (summarised by e.g. Todorov and Jordan 2002) suggests that these functions are not strictly separated in human movements. Control strategies utilising a unified plan-and-execute paradigm have been proposed, such as optimal feedback control (Todorov and Jordan 2002) and its extension with learned dynamics (Mitrovic et al. 2010), but they are computationally costly and cumbersome to train for complex models with nonlinear muscle properties, including activation dynamics. Regardless, understanding how muscle properties affect planning and execution separately can further comprehension of neuromuscular coordination as a whole. Furthermore, while the reaches in our study track a straight line with a bell-shaped speed profile, the model is expected to be able to execute any reaching plan provided it is feasible in terms of positions, velocities and accelerations required. However, significant changes to the desired trajectories, including continuous updating throughout movement, likely requires re-tuning feedback gains and affects co-contraction levels.

The simplifications in the model and the control strategy leave out a number of features of the neuromuscular control system. We excluded muscle elasticity because its impact only becomes noticeable in more demanding tasks than explored presently. The feedback in our controller is conceptual rather than representative of any neural control loop; we opted to maximise tractability by avoiding additional delays and coordinate system conversions inherent in potential feedback pathways. For example, visually guided cortical control of reaching is subject to processing and transmission delays up to 200–300 ms (Miall et al. 1993), which could obscure any effect seen due to activation delays. Spinal reflexes are faster, but they require that the desired trajectory is specified in sensory coordinates such as muscle length, speed, or force, which becomes increasingly complicated with non-linear muscles and moment arms. Another key feature of neuromuscular control not modelled is the rate coding and motor unit recruitment pattern of muscle excitation (e.g. Enoka and Duchateau 2017) which is beyond the current scope because we seek to understand how gross (time-averaged) activation effects influence control. However, rate coding could be integrated in a future model to explore effects of varying motor unit properties.

## 5. Conclusion

We investigated how intrinsic muscle properties interact to influence the neuromuscular control of reaching. Our simulations suggest that high reaching performance can be achieved with physiologically realistic muscle models. However, the maintenance of movement characteristics with increasingly realistic muscle properties came at the cost of longer control prediction times and increased co-contraction. We therefore propose that simple muscle models are sufficient when only the behavioural outcome is of interest. However, caution should be used when addressing questions related to neuromuscular control, as high-order activation models, needed to capture realistic muscle behaviour, can affect the key features of the control problem.

## Endnote

1. For simplicity, we refer to horizontal adduction/abduction of the shoulder also as flexion/extension.
